# Effectiveness of the combination elvitegravir/cobicistat/tenofovir/emtricitabine (EVG/COB/TFV/FTC) plus darunavir among treatment-experienced patients in clinical practice: a multicentre cohort study

**DOI:** 10.1186/s12981-020-00302-2

**Published:** 2020-07-20

**Authors:** Inés Suárez-García, Cristina Moreno, Marta Ruiz-Algueró, María Jesús Pérez-Elías, Marta Navarro, Marcos Díez Martínez, Pompeyo Viciana, Laura Pérez-Martínez, Miguel Górgolas, Concha Amador, Miguel Alberto de Zárraga, Inma Jarrín, the Cohort of the Spanish HIV/AIDS Research Network (CoRIS), Santiago Moreno, Inma Jarrín, David Dalmau, Maria Luisa Navarro, Maria Isabel González, Federico Garcia, Eva Poveda, Jose Antonio Iribarren, Félix Gutiérrez, Rafael Rubio, Francesc Vidal, Juan Berenguer, Juan González, M Ángeles Muñoz-Fernández, Inmaculada Jarrin, Belén Alejos, Cristina Moreno, Carlos Iniesta, Luis Miguel Garcia Sousa, Nieves Sanz Perez, Marta Rava, M Ángeles Muñoz-Fernández, Irene Consuegra Fernández, Esperanza Merino, Gema García, Irene Portilla, Iván Agea, Joaquín Portilla, José Sánchez-Payá, Juan Carlos Rodríguez, Lina Gimeno, Livia Giner, Marcos Díez, Melissa Carreres, Sergio Reus, Vicente Boix, Diego Torrús, Ana López Lirola, Dácil García, Felicitas Díaz-Flores, Juan Luis Gómez, María del Mar Alonso, Ricardo Pelazas, Jehovana Hernández, María Remedios Alemán, María Inmaculada Hernández, Víctor Asensi, Eulalia Valle, María Eugenia Rivas Carmenado, Tomas Suarez-Zarracina Secades, Laura Pérez Is, Rafael Rubio, Federico Pulido, Otilia Bisbal, Asunción Hernando, Lourdes Domínguez, David Rial Crestelo, Laura Bermejo, Mireia Santacreu, José Antonio Iribarren, Julio Arrizabalaga, María José Aramburu, Xabier Camino, Francisco Rodríguez-Arrondo, Miguel Ángel von Wichmann, Lidia Pascual Tomé, Miguel Ángel Goenaga, Mª Jesús Bustinduy, Harkaitz Azkune, Maialen Ibarguren, Aitziber Lizardi, Xabier Kortajarena, Mª Pilar Carmona Oyaga, Maitane Umerez Igartua, Félix Gutiérrez, Mar Masiá, Sergio Padilla, Andrés Navarro, Fernando Montolio, Catalina Robledano, Joan Gregori Colomé, Araceli Adsuar, Rafael Pascual, Marta Fernández, Elena García, José Alberto García, Xavier Barber, Roberto Muga, Arantza Sanvisens, Daniel Fuster, Juan Berenguer, Juan Carlos López Bernaldo de Quirós, Isabel Gutiérrez, Margarita Ramírez, Belén Padilla, Paloma Gijón, Teresa Aldamiz-Echevarría, Francisco Tejerina, Francisco José Parras, Pascual Balsalobre, Cristina Diez, Leire Pérez Latorre, Chiara Fanciulli, Francesc Vidal, Joaquín Peraire, Consuelo Viladés, Sergio Veloso, Montserrat Vargas, Montserrat Olona, Anna Rull, Esther Rodríguez-Gallego, Verónica Alba, Alfonso Javier Castellanos, Miguel López-Dupla, Marta Montero Alonso, José López Aldeguer, Marino Blanes Juliá, María Tasias Pitarch, Iván Castro Hernández, Eva Calabuig Muñoz, Sandra Cuéllar Tovar, Miguel Salavert Lletí, Juan Fernández Navarro, Juan González-Garcia, Francisco Arnalich, José Ramón Arribas, Jose Ignacio Bernardino de la Serna, Juan Miguel Castro, Luis Escosa, Pedro Herranz, Victor Hontañón, Silvia García-Bujalance, Milagros García López-Hortelano, Alicia González-Baeza, Maria Luz Martín-Carbonero, Mario Mayoral, Maria Jose Mellado, Rafael Esteban Micán, Rocio Montejano, María Luisa Montes, Victoria Moreno, Ignacio Pérez-Valero, Berta Rodés, Talia Sainz, Elena Sendagorta, Natalia Stella Alcáriz, Eulalia Valencia, José Ramón Blanco, José Antonio Oteo, Valvanera Ibarra, Luis Metola, Mercedes Sanz, Laura Pérez-Martínez, Piedad Arazo, Gloria Sampériz, David Dalmau, Angels Jaén, Montse Sanmartí, Mireia Cairó, Javier Martinez-Lacasa, Pablo Velli, Roser Font, Mariona Xercavins, Noemí Alonso, Maria Rivero Marcotegui, Jesús Repáraz, María Gracia Ruiz de Alda, María Teresa de León Cano, Beatriz Pierola Ruiz de Galarreta, María José Amengual, Gemma Navarro, Manel Cervantes Garcia, Sonia Calzado Isbert, Marta Navarro Vilasaro, Ignacio de los Santos, Jesus Sanz Sanz, Ana Salas Aparicio, Cristina Sarria Cepeda, Lucio Garcia-Fraile Fraile, Enrique Martín Gayo, Santiago Moreno, José Luis Casado Osorio, Fernando Dronda Nuñez, Ana Moreno Zamora, Maria Jesús Pérez Elías, Carolina Gutiérrez, Nadia Madrid, Santos del Campo Terrón, Sergio Serrano Villar, Maria Jesús Vivancos Gallego, Javier Martínez Sanz, Usua Anxa Urroz, Tamara Velasco, Enrique Bernal, Alfredo Cano Sanchez, Antonia Alcaraz García, Joaquín Bravo Urbieta, Angeles Muñoz Perez, Maria Jose Alcaraz, Maria del Carmen Villalba, Federico García, José Hernández Quero, Leopoldo Muñoz Medina, Marta Alvarez, Natalia Chueca, David Vinuesa García, Clara Martinez-Montes, Carlos Guerrero Beltran, Adolfo de Salazar Gonzalerz, Ana Fuentes Lopez, Montserrat Raposo Utrilla, Jorge Del Romero, Carmen Rodríguez, Teresa Puerta, Juan Carlos Carrió, Mar Vera, Juan Ballesteros, Oskar Ayerdi, Antonio Antela, Elena Losada, Melchor Riera, María Peñaranda, María Leyes, Mª Angels Ribas, Antoni A Campins, Carmen Vidal, Francisco Fanjul, Javier Murillas, Francisco Homar, Jesús Santos, Crisitina Gómez Ayerbe, Isabel Viciana, Rosario Palacios, Carmen Pérez López, Carmen Maria Gonzalez-Domenec, Pompeyo Viciana, Nuria Espinosa, Luis Fernando López-Cortés, Daniel Podzamczer, Arkaitz Imaz, Juan Tiraboschi, Ana Silva, María Saumoy, Paula Prieto, Esteban Ribera, Adrian Curran, Julián Olalla Sierra, Javier Pérez Stachowski, Alfonso del Arco, Javier de la torre, José Luis Prada, José María García de Lomas Guerrero, Onofre Juan Martínez, Francisco Jesús Vera, Lorena Martínez, Josefina García, Begoña Alcaraz, Amaya Jimeno, Angeles Castro Iglesias, Berta Pernas Souto, Alvaro Mena de Cea, Josefa Muñoz, Miren Zuriñe Zubero, Josu Mirena Baraia-Etxaburu, Sofía Ibarra Ugarte, Oscar Luis Ferrero Beneitez, Josefina López de Munain, Mª Mar Cámara López, Mireia de la Peña, Miriam Lopez, Iñigo Lopez Azkarreta, Carlos Galera, Helena Albendin, Aurora Pérez, Asunción Iborra, Antonio Moreno, Maria Angustias Merlos, Asunción Vidal, Marisa Meca, Concha Amador, Francisco Pasquau, Javier Ena, Concha Benito, Vicenta Fenoll, Concepcion Gil Anguita, Jose Tomas Algado Rabasa, Inés Suárez-García, Eduardo Malmierca, Patricia González-Ruano, Dolores Martín Rodrigo, Mª Pilar Ruiz Seco, Mohamed Omar Mohamed-Balghata, María Amparo Gómez Vidal, Miguel Alberto de Zarraga, Vicente Estrada Pérez, Maria Jesus Téllez Molina, Jorge Vergas García, Juncal Pérez-Somarriba Moreno, Miguel Górgolas, Alfonso Cabello, Beatriz Álvarez, Laura Prieto, José Sanz Moreno, Alberto Arranz Caso, Cristina Hernández Gutiérrez, María Novella Mena, María José Galindo Puerto, Ramón Fernando Vilalta, Ana Ferrer Ribera, Antonio Rivero Román, Antonio Rivero Juárez, Pedro López López, Isabel Machuca Sánchez, Mario Frias Casas, Angela Camacho Espejo, Miguel Cervero Jiménez, Rafael Torres Perea, Juan A Pineda, Pilar Rincón Mayo, Juan Macias Sanchez, Nicolas Merchante Gutierrez, Luis Miguel Real, Anais Corma Gomez, Marta Fernandez Fuertes, Alejandro Gonzalez-Serna, Eva Poveda, Alexandre Pérez, Manuel Crespo, Luis Morano, Celia Miralles, Antonio Ocampo, Guillermo Pousada

**Affiliations:** 1 Department of Internal Medicine, Infectious Diseases Group, Hospital Universitario Infanta Sofía, Paseo de Europa, 34. San Sebastián de los Reyes, 28702 Madrid, Spain; 2 FIIB HUIS HHEN, Madrid, Spain; 3 Universidad Europea, Madrid, Spain; 4 Centro Nacional de Epidemiología, Instituto de Salud Carlos III, Madrid, Spain; 5 Hospital Universitario Ramón y Cajal, Madrid, Spain; 6 Corporació Sanitària Parc Taulí, Barcelona, Spain; 7 Hospital General Universitario de Alicante, Alicante, Spain; 8 Hospital Virgen del Rocío, Sevilla, Spain; 9 Hospital San Pedro Centro de Investigación Biomédica de La Rioja (CIBIR), Logroño, La Rioja Spain; 10 Hospital Universitario Fundación Jiménez Díaz, Madrid, Spain; 11 Hospital de la Marina Baixa, La Vila Joiosa, Spain; 12 Hospital San Agustín, Avilés, Spain

**Keywords:** HIV infection, Highly active antiretroviral therapy, Cohort studies, Darunavir

## Abstract

**Background:**

The aim of this study was to investigate the effectiveness and tolerability of the combination elvitegravir/cobicistat/tenofovir/emtricitabine plus darunavir (EVG/COB/TFV/FTC + DRV) in treatment-experienced patients from the cohort of the Spanish HIV/AIDS Research Network (CoRIS).

**Methods:**

Treatment-experienced patients starting treatment with EVG/COB/TFV/FTC + DRV during the years 2014–2018 and with more than 24 weeks of follow-up were included. TFV could be administered either as tenofovir disoproxil fumarate or tenofovir alafenamide. We evaluated virological response, defined as viral load (VL) < 50 copies/ml and < 200 copies/ml at 24 and 48 weeks after starting this regimen, stratified by baseline VL (< 50 or ≥ 50 copies/ml at the start of the regimen).

**Results:**

We included 39 patients (12.8% women). At baseline, 10 (25.6%) patients had VL < 50 copies/ml and 29 (74.4%) had ≥ 50 copies/ml. Among patients with baseline VL < 50 copies/ml, 85.7% and 80.0% had VL < 50 copies/ml at 24 and 48 weeks, respectively, and 100% had VL < 200 copies/ml at 24 and 48 weeks. Among patients with baseline VL ≥ 50 copies/ml, 42.3% and 40.9% had VL < 50 copies/ml and 69.2% and 68.2% had VL < 200 copies/ml at 24 and 48 weeks. During the first 48 weeks, no patients changed their treatment due to toxicity, and 4 patients (all with baseline VL ≥ 50 copies/ml) changed due to virological failure.

**Conclusions:**

EVG/COB/TFV/FTC + DRV was well tolerated and effective in treatment-experienced patients with undetectable viral load as a simplification strategy, allowing once-daily, two-pill regimen with three antiretroviral drug classes. Effectiveness was low in patients with detectable viral loads.

## Background

Treatment adherence is crucial for the effectiveness of antiretroviral therapy (ART) among HIV-infected patients. Adherence could be impaired with antiretroviral regimens that entail multiple pills or more than once-daily dosing [1]; this is a frequent situation among patients with drug-resistant HIV. Simplification of complex ART regimens is an important strategy for reducing the number of pills or doses per day and is intended to improve patients’ adherence and quality of life without compromising treatment effectiveness [2].

The fixed-dose combinations elvitegravir/cobicistat/tenofovir disoproxil fumarate/emtricitabine (EVG/COB/TDF/FTC, Stribild^®^) and elvitegravir/cobicistat/tenofovir alafenamide/emtricitabine (EVG/COB/TAF/FTC, Genvoya^®^) have been extensively used for the treatment of HIV-infected adults [3, 4]. The association of one of those fixed-dose combinations with darunavir 800 mg (DRV) would allow a once-daily, two-pill regimen that includes three classes of antiretroviral drugs and could be used to treat selected patients with drug-resistant HIV. This is an off-label indication.

With these combinations, COB, which boosts EVG, would also allow boosting of DRV [5]. However, pharmacokinetic studies evaluating drug levels in patients receiving EVG/COB/TDF/FTC plus DRV have found conflicting results. While some studies have found lower levels of DRV and EVG in patients receiving EVG/COB/TDF/FTC + DRV compared to those receiving each drug as a separate component (DRV being boosted either with COB or ritonavir) [6, 7], others found similar DRV and EVG levels in patients receiving this combination compared to those receiving each separate drug [8, 9]. All these studies involved a very low number of patients (between 5 and 24).

The efficacy of EVG/COB/TAF/FTC + DRV in virologically suppressed patients was demonstrated in an open-label clinical trial, which showed that switching to EVG/COB/TAF/FTC + DRV was non-inferior to maintaining the previous ART at 24 weeks and superior at 48 weeks [10]. However, in spite of the results of this clinical trial and the potential advantages of this combination for ART simplification, there is very little evidence in real-life clinical practice. Only three single-centre cohort studies analysing EVG/COB/TDF/FTC + DRV have been published, involving a very low number of patients (21, 10, and 17 patients) and with limited follow-up [5, 6, 11].

Given the very scarce evidence with the combinations EVG/COB/TDF/FTC + DRV and EVG/COB/TAF/FTC + DRV in clinical practice, the conflicting results of pharmacokinetic studies, and the potential advantages of these combinations as a simplification strategy, we designed a study to describe the use of elvitegravir/cobicistat/tenofovir (administered either as disoproxil fumarate or alafenamide)/emtricitabine plus darunavir 800 mg (EVG/COB/TFV/FTC + DRV) and analyse its effectiveness and tolerability in the multicentre Cohort of the Spanish HIV/AIDS Research Network (CoRIS). The specific objectives of the study were: (1) to describe the patients initiating EVG/COB/TFV/FTC + DRV and the reasons for starting this regimen, (2) to describe the toxicity of this regimen among patients who stopped it due to adverse events, and (3) to describe the effectiveness of EVG/COB/TFV/FTC + DRV in terms of viral suppression and change in CD4 count at 24 and 48 weeks after starting this regimen.

## Methods

Patients were selected from the Cohort of the Spanish HIV/AIDS Research Network (CoRIS), which has been described in detail elsewhere [12, 13]. CoRIS is a prospective multicentre cohort of adult HIV-positive treatment-naïve patients, recruited from 46 centres from 13 Autonomous Regions in the Spanish public healthcare system. Since January 2004 to the last update in November 2018, 15509 patients have been recruited in CoRIS.

We included all treatment-experienced patients who started treatment with EVG/COB/TFV/FTC + DRV from January 2014 to November 2018 and who had more than 24 weeks of follow-up. TFV could be administered either as tenofovir disoproxil fumarate or tenofovir alafenamide.

We collected information on the following variables: age, sex, mode of HIV transmission (men who have sex with men [MSM], heterosexual, injecting drug user [IDU], other/unknown), country of origin (Spanish, foreign-born), CD4 count and viral load at the start of EVG/COB/TFV/FTC + DRV and at 24 and 48 weeks after starting this regimen (± 12 weeks), reasons for stopping the previous treatment and EVG/COB/TFV/FTC + DRV (simplification, virological failure, toxicity, interactions, other, unknown) and regimen to which it was switched, previous antiretroviral regimens, number of pills and doses per day with the previous antiretroviral regimen, and time since the start of the first ART.

Descriptive analyses were carried out using frequency tables for categorical variables and median and interquartile range (IQR) for continuous variables. For the analysis of treatment effectiveness, the primary endpoint was the proportion of patients with virological response, defined as viral load < 50 copies/ml at 24 and 48 weeks after starting EVG/COB/TFV/FTC + DRV. Since the threshold of 200 copies/ml is used in several guidelines to define virologic failure [14, 15], we also analysed a secondary endpoint of virological response defined as viral load < 200 copies at 24 and 48 weeks. We also evaluated the median change in CD4 count at 24 and 48 weeks, and the proportion of patients stopping their treatment due to virological failure and due to adverse events. Results were stratified by baseline viral load (< 50 or ≥ 50 copies/ml): baseline was defined as the time of the initiation of EVG/COB/TFV/FTC + DRV. For the endpoints of virological response and change in CD4 count, we performed an intention to treat analysis: therefore, once the treatment was started, all patients were assumed to remain on it and subsequent changes were ignored.

Statistical analyses were performed in Stata software (version 14.0; Stata Corporation, College Station, Texas, USA). All patients signed informed consent forms. The study was approved by the Ethics Committee of Instituto de Salud Carlos III (Madrid).

## Results

During the study period, 39 patients from 11 centres switched their ART to the regimen EVG/COB/TFV/FTC + DRV. Patients’ demographic and clinical characteristics are shown in Table 1. At the start of the regimen, tenofovir was administered as TDF in 16 and as TAF in 23 patients. During follow-up, eight of the 16 patients who started the regimen with TDF switched TDF to TAF as part of the same regimen.

**Table 1:** Patients’ characteristics at the start of EVG/COB/TFV/FTC + DRV (n = 39)

Age, median (IQR), years	42 (34–50)
Female	5 (12.8)
Mode of transmission	
Men who have sex with men	19 (48.7)
Heterosexual	13 (33.3)
Injecting drug user	5 (12.8)
Other/unknown	2 (5.1)
Geographic origin	
Spanish	14 (33.3)
Foreign-born	24 (61.5)
Unknown	1 (2.4)
Viral load, median (IQR), copies/ml	379 (40–12,000)
CD4 count, median (IQR), cells/microl	437 (108–740)
Viral load	
< 50 copies/ml	10 (25.6)
≥ 50 copies/ml	29 (74.4)
Years since starting ART, median (IQR)	5.3 (2.5–7.5)
Number of prior regimens, median (IQR)	3 (2–6)
Previous ART regimen	
ART daily pill burden, median (IQR)	2 (1–5)
At least 3 pills per day	17 (43.6)
At least twice daily ART dosing	11 (28.2)
Reasons for switching to EVG/COB/TFV/FTC + DRV	
Virologic failure	14 (35.9)
Simplification	10 (25.6)
Toxicity	4 (10.3)
Non-adherence	2 (5.1)
Unknown	9 (23.1)

Values are expressed as n/total (%) unless stated otherwise

*IQR* Interquartile range

At baseline, 10 (25.6%) patients were virologically suppressed and 29 (74.4%) patients had detectable viral load. The most frequent reasons for changing to this regimen were virological failure and treatment simplification (Table 1).

The patients received EVG/COB/TFV/FTC + DRV for a median of 391 (IQR: 205 to 514) days. Outcomes at 24 and 48 weeks are shown in Table 2.

**Table 2:** Outcomes of patients at 24 and 48 weeks after starting EVG/COB/TFV/FTC + DRV, stratified by viral load at the start of the regimen

Outcome	24 weeks	48 weeks
Virological response < 50 copies/ml		
Viral load < 50 copies/ml	6/7 (85.7)	4/5 (80.0)
Viral load ≥ 50 copies/ml	11/26 (42.3)	9/22 (40.9)
Virological response < 200 copies/ml		
Viral load < 50 copies/ml	7/7 (100.0)	5/5 (100.0)
Viral load ≥ 50 copies/ml	18/26 (69.2)	15/22 (68.2)
CD4 change, cells/microl: median (IQR)		
Viral load < 50 copies/ml	29 (14–48)	8 (− 85–50)
Viral load ≥ 50 copies/ml	− 6 (− 134–107)	− 16 (− 116–77)
Patients stopping the regimen for any reason		
Viral load < 50 copies/ml	0/10 (0)	2/10 (20.0)
Viral load ≥ 50 copies/ml	8/29 (27.6)	13/29 (44.8)
Patients stopping the regimen due to treatment failure		
Viral load < 50 copies/ml	0/10 (0)	0/10 (0)
Viral load ≥ 50 copies/ml	4/29 (13.8)	4/29 (13.8)
Patients stopping the regimen due to toxicity		
Viral load < 50 copies/ml	0/10 (0)	0/10 (0)
Viral load ≥ 50 copies/ml	0/29 (0)	0/29 (0)

Values are expressed as n/total (%) unless stated otherwise

*IQR* Interquartile range

Among virologically suppressed patients at baseline, 85.7% and 80.0% of the patients achieved viral load < 50 copies/ml at 24 and 48 weeks, respectively. However, among patients with detectable baseline viral load, only 42.3% and 40.9% achieved these endpoints, respectively. The percentages of patients achieving viral load < 200/ml at 24 and 48 weeks were higher for both groups and reached 100% among the patients who were virologically suppressed at baseline.

The number of patients who changed their treatment during the first 24 and 48 weeks, stratified by baseline viral load, is shown in Table 2. Among the two virologically suppressed patients at baseline who changed their treatment during the first 48 weeks, the reasons were simplification in 1 patient and unknown in 1 patient. Among patients with baseline detectable viral load, the reasons for changing the regimen during the first 24 weeks were failure in 4, simplification in 3, and non-adherence in 1 patient, and the reasons for changing the regimen during the first 48 weeks were simplification in 7 patients, failure in 4 patients, nonadherence in 1 patient, and unknown in 1 patient.

## Discussion

This study has analysed the largest cohort published to date showing results from “real-world” clinical practice with the regimen EVG/COB/TFV/FTC + DRV. In our study, EVG/COB/TFV/FTC + DRV was well tolerated and effective in treatment-experienced patients with baseline undetectable viral load as a simplification strategy, allowing a once-daily, two-pill regimen with three antiretroviral drug classes. However, effectiveness was low in patients with baseline detectable viral loads.

There is only one open-label clinical trial that assessed the efficacy of this regimen, which analysed 135 treatment-experienced, virologically suppressed patients who were randomized to continue their previous ART or change to EVG/COB/TAF/FTC + DRV. The study found that this combination had high efficacy (96.6%) and was noninferior to maintaining the previous ART at 24 weeks and superior at 48 weeks [10]. However, it is necessary to contrast the findings from clinical trials with real-world data, such as those obtained from cohort studies: clinical trials include selected patients that are often different from those of the population who will receive those treatments (such as lower number of patients with severe immunosuppression, elderly patients, or those with co-morbidities), and provide more intensive monitoring to the patients (which could influence adherence to treatment and detection of adverse effects), limiting the generalizability of their findings [16, 17].

Regarding real-world data, the evidence for the effectiveness of this regimen is very limited: only three cohort studies have been published analysing EVG/COB/TDF/FTC + DRV, all from single centres, involving a very low number of patients, and analysing different endpoints. The effectiveness was high in the three studies: Naccarato et al. evaluated 21 treatment-experienced patients (29% of which were virologically suppressed before the switch) who received EVG/COB/TDF/FTC + DRV: at week 48, 14 (67%) patients had undetectable viral load, 1 had virologic failure (> 40 copies/ml) and 6 had stopped the treatment or had no viral load data [5]. Harris et al. evaluated the simplification to the same regimen in 10 virologically suppressed patients: 8 of them had viral loads < 40 copies/ml at weeks 24 and 48 and all of them had viral loads < 200 copies/ml up to week 48 [6]. The last study by Diaz et al. found that 15 (88%) of 17 patients (naïve and treatment-experienced) had viral loads < 50 copies/ml at week 24 [11].

In our study, the effectiveness of EVG/COB/TFV/FTC + DRV was high in patients who were virologically suppressed at baseline: 85.7% and 80.0% of the patients had viral loads < 50 copies at 24 and 48 weeks, respectively, and 100% of the patients had viral loads < 200 copies/ml at 24 and 48 weeks. These results are comparable to the studies mentioned above. However, the effectiveness was much lower for patients who had detectable viral loads at baseline. These results suggest that this regimen has high effectiveness as a switch strategy for virologically suppressed patients, but it should not be used in patients with virological failure if other alternatives exist. Overall, the treatment was well tolerated in both groups, as none of the patients discontinued their treatment due to toxicity. The treatment also allowed to decrease the pill burden in 43.6% of the patients and the number of doses per day in 28.2%.

For the combination EVG/COB/TFV/FTC + DRV, our patients were receiving tenofovir either as alafenamide or as disoproxil fumarate. Also, as EVG/COB/TAF/FTC was commercialized in Spain in May 2016, many patients who were receiving EVG/COB/TDF/FTC changed their treatment to EVG/COB/TAF/FTC in order to minimize the risk of renal and bone toxicity, as did 8 of our patients. Given that the treatment with EVG/COB/TAF/FTC has shown non-inferiority compared to EVG/COB/TDF/FTC in clinical trials [3, 4], we have analysed our results combining both treatments. To ensure that this was an adequate strategy, we repeated our analysis stratifying by treatment with TAF or TDF: the results were not changed (data not shown).

Our study has the limitation of low sample size. Also, since CoRIS does not routinely record resistance testing in pre-treated patients, we could not describe accumulated resistance mutations or those arising after failure with this regimen. Another limitation is that we cannot give information on patients’ adherence as CoRIS does not routinely record this variable. However, this is the largest cohort published to date analysing this treatment regimen, with a reasonable follow-up time, and it gives information from routine clinical practice for a combination that could be potentially useful for treatment simplification and has very little published evidence. Our strengths include the use of a multicentre cohort with rigorous quality control and which has clinical and demographic characteristics that are similar to the ones from the general population reported by the National HIV Surveillance System [18].

## Conclusion

The combination EVG/COB/TFV/FTC + DRV is an effective, well tolerated strategy for treatment simplification in virologically suppressed patients. This treatment is not suitable, however, for treatment-experienced individuals with detectable viral loads, given the low efficacy in this group of patients.

## Data Availability

The authors confirm that, for approved reasons, some access restrictions apply to the data underlying the findings. Data cannot be made publicly available because they are owned by a third party, the HIV/AIDS Research Network (RIS), and because participants agreed that data would only be used for research projects by RIS or those projects approved by its Executive and Scientific Committee. Interested readers may send requests for the data to proyectoscoris@gmail.com. Requests will be assessed by the Executive and Scientific Committee.

## Abbreviations

ART: Antiretroviral therapy
CoRIS: Cohort of the Spanish HIV/AIDS Research Network
DRV: Darunavir
EVG/COB/TAF/FTC: Elvitegravir/cobicistat/tenofovir alafenamide/emtricitabine
EVG/COB/TDF/FTC: Elvitegravir/cobicistat/tenofovir disoproxil fumarate/emtricitabine
EVG/COB/TFV/FTC: Elvitegravir/cobicistat/tenofovir/emtricitabine
IDU: Injecting drug user
IQR: Interquartile range
MSM: Men who have sex with men
VL: Viral load
